# Alternative Oxidase: A Potential Target for Controlling Aflatoxin Contamination and Propagation of *Aspergillus flavus*

**DOI:** 10.3389/fmicb.2020.00419

**Published:** 2020-03-17

**Authors:** Fei Tian, Sang Yoo Lee, So Young Woo, Hyang Sook Chun

**Affiliations:** Food Toxicology Laboratory, Advanced Food Safety Research Group, BK21 Plus, School of Food Science and Technology, Chung-Ang University, Anseong, South Korea

**Keywords:** aflatoxin, alternative oxidase, mitochondria, respiration, antiaflatoxigenic activity

## Abstract

Aflatoxins are among the most hazardous natural cereal contaminants. These mycotoxins are produced by *Aspergillus* spp. as polyketide secondary metabolites. Aflatoxigenic fungi including *A. flavus* express the alternative oxidase (AOX), which introduces a branch in the cytochrome-based electron transfer chain by coupling ubiquinol oxidation directly with the reduction of O_2_ to H_2_O. AOX is closely associated with fungal pathogenesis, morphogenesis, stress signaling, and drug resistance and, as recently reported, affects the production of mycotoxins such as sterigmatocystin, the penultimate intermediate in aflatoxin B_1_ biosynthesis. Thus, AOX might be considered a target for controlling the propagation of and aflatoxin contamination by *A. flavus*. Hence, this review summarizes the current understanding of fungal AOX and the alternative respiration pathway and the development and potential applications of AOX inhibitors. This review indicates that AOX inhibitors, either alone or in combination with current antifungal agents, are potentially applicable for developing novel, effective antifungal strategies. However, considering the conservation of AOX in fungal and plant cells, a deeper understanding of fungal alternative respiration and fungal AOX structure is needed, along with effective fungal-specific AOX inhibitors.

## Introduction

Aflatoxin contamination is a food safety concern worldwide, affecting both the marketability and safety of multiple food crops such as maize, peanuts, and tree nuts (Kumar et al., 2016). Aflatoxins are primarily produced by *Aspergillus* spp., including *A. flavus*, as polyketide secondary metabolites. These opportunistic fungi are commonly detected as contaminants in cereal crops at both the pre- and post-harvest stages (Umesha et al., 2017). Although aflatoxigenic fungi more commonly grow in tropical and sub-tropical climates, aflatoxin contamination has always been a global concern owing to globalized trade; moreover, zones with a perennial aflatoxin contamination risk have expanded owing to climate change (Marroquín-Cardona et al., 2014; Baranyi et al., 2015). Current methods of preventing aflatoxin contamination in food cereals primarily depend on the continued application of synthetic fungicides, which, although effective, target a limited number of cellular phenomena and also have side effects such as toxicity among humans and other animals, environmental pollution, and development of resistance in phytopathogens (Panáček et al., 2009). Therefore, new antifungal and antiaflatoxigenic strategies are urgently needed.

The respiratory chain is an effective target for fungicides to control fungal contamination in food crops. The presence of fungal-specific respiration components and the recent discovery of the association between respiration and pathogenesis in several phytopathogenic species have fostered the development of new mitochondria-targeted fungicides. However, the emergence of rapid resistance to currently used inhibitors, toxicity concerns raised from the conservation of the respiratory machinery in eukaryotes, and the limited understanding of the physiological roles of mitochondria have largely deterred the application of respiration inhibitors as fungicides.

**FIGURE 1:**
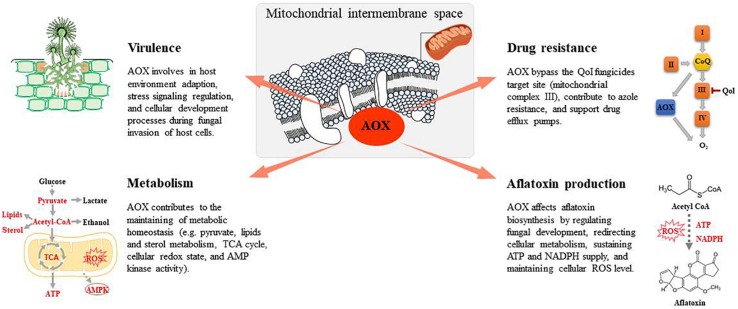
Fungal alternative oxidase (AOX) functions associated with antifungal studies.

The alternative oxidase (AOX) is an integral monotopic membrane protein localized on the matrix side of the inner mitochondrial membrane (Figure 1). The enzyme is ubiquitous in the plant kingdom and is present in numerous pathogenic and agriculturally important fungi including *Aspergillus* spp., *Candida* spp., *Hansenula* spp., and *Magnaporthe* spp. (Joseph-Horne et al., 2001; Tudella et al., 2004). AOX introduces a branch in the cytochrome-based electron transfer chain by coupling ubiquinol oxidation directly with the reduction of O_2_ to H_2_O. Consequently, fewer protons migrate across the inner mitochondrial membrane, generating a proton gradient, leading to markedly lesser ATP production through oxidative phosphorylation (Joseph-Horne et al., 2001; Young et al., 2013). AOX activity is associated with reactive oxygen species (ROS) control, metabolic homeostasis, cellular energy demand, the redox state, and the stress response (Li et al., 2011; Vanlerberghe, 2013). Furthermore, AOX affects mycotoxin production, such as sterigmatocystin, which is the penultimate intermediate in aflatoxin B_1_ biosynthesis (Leiter et al., 2016; Molnár et al., 2018a). As AOX is absent in mammals, it has been investigated as a potential drug target for pathogenic fungi (Tudella et al., 2004; Ebiloma et al., 2019). Genomic sequence analyses have predicted the presence of at least one AOX in each complete *Aspergillus* genome sequence including *A. clavatus*, *A. flavus*, *A. fumigatus*, *A. nidulans*, and *A. niger* (Table 1). AOX exhibits high levels of conservation and genome synteny across *Aspergillus* spp. (Li et al., 2011).

**TABLE 1:** The existence and reported functions of alternative oxidase (AOX) in *Aspergillus spp.*

Species	Number of AOX	GenBank accession number	Reported functions	References
*Aspergillus bombycis*	3	XM_022535135, XM_022530731, XM_022527953	No report	−^1)^
*A. caelatus*	3	XM_032076675, XM_032072264, XM_032065812	No report	–
*A. clavatus*	2	XM_001273619, XM_001272692	No report	–
*A. flavus*	2	XM_002377259, XM_002374976	No report	–
*A. fumigatus*	1	XM_744544	Oxidative stress resistance; oxygen uptake; pathogenesis; macrophage susceptibility.	Magnani et al., 2007; Grahl et al., 2012
*A. nidulans*	1	XM_654611	Sterigmatocystin production regulation; oxygen uptake; conidiospores production and conidia viability.	Leiter et al., 2016; Molnár et al., 2018a
*A. niger*	2	XM_001394435, XM_001394775	Heat, oxidative and osmotic stress response; metabolic flow regulation; energy metabolism.	Honda et al., 2012; Hou et al., 2018
*A. nomius*	2	XM_015556206, XM_015548398	No report	–
*A. oryzae*	2	XM_001819459, XM_023232744	No report	–
*A. terreus*	2	XM_001216061, XM_001215177	Oxygen uptake; limiting ROS generation; defense against oxidative stress; metabolic flow regulation.	Pérez-Sánchez et al., 2017; Molnár et al., 2018b

*^1)^Not applicable.*

Although AOX is a promising target for the development of novel antifungal strategies, further studies are required to understand the physiological function of AOX and its association with fungal pathogenesis, morphogenesis, stress signaling, drug resistance, environment adaption, and secondary metabolism. Hence, this review addresses these issues by summarizing the current understanding of AOX and the alternative respiration pathway in pathogenic and agriculturally important fungi, and progress in studies on AOX inhibitors as antifungal agents.

## AOX and Fungal Virulence

Similar to other facultative parasites, the virulence of *A. flavus* is multifactorial and is closely connected with the cellular development, secondary metabolism, adaption to stress conditions, and interaction with host defense molecules (Amaike and Keller, 2011). An intact and functional electron transport system is important for fungal virulence (Chatre and Ricchetti, 2014). Numerous pathogenic fungi depend on oxidative phosphorylation for virulence. Respiratory activity is crucial for energy generation and for adaptation to the host environment. For example, through the life cycle of phytopathogen *Moniliophthora perniciosa*, the causal agent of the witches’ broom disease of cocoa, AOX is overexpressed in the biotrophic phase, when live cocoa cells produce large amounts of nitric oxide, which serve as respiration inhibitors. Ruiz et al. (2011) reported that in *Paracoccidioides brasiliensis*, AOX is important in the fungal defense against oxidative stress imposed by immune cells and is relevant to the virulence of this human pathogenic fungus. Akhter et al. (2003) reported that AOX contributes to the virulence composite of *Cryptococcus neoformans*, potentially by improving survival within phagocytic cells. Furthermore, defects in the electron transport system in *C. albicans* affect the hyphal morphological switch, an important determinant of virulence (McDonough et al., 2002; Khamooshi et al., 2014).

Furthermore, the role of AOX in stress signaling potentially contributes to the survival of fungal pathogens in the host environment. AOX is suggested to contribute to the regulation of the ROS balance, maintenance of the redox state, and response to various types of stress (Millenaar and Lambers, 2008). Oxidative stress specifically inhibits some key metabolic enzymes including glyceraldehyde-3-phosphate dehydrogenase (Li et al., 2011) and aconitase (Murakami and Yoshino, 1997), which are essential for fungal primary metabolism. Owing to the non-proton pumping nature of AOX, the fungal alternative respiration pathway is suggested to reduce ROS generation. AOX is apparently induced under oxidative stress to minimize the negative effects caused by excess ROS; however, this in turn led to major metabolic changes in fungal cells upon a reduction in the ATP supply.

Fungal respiration and virulence may also be associated with cellular remodeling. For example, Grahl et al. (2015) reported that in *C. albicans*, the disease-associated morphological switch from yeast to hyphal growth is affected by mitochondrial functions, as mitochondrial inhibitors potently suppress the Ras1-Cyr1-PKA pathway, a major regulator of *C. albicans* morphogenesis, biofilm formation, and white-opaque switching. Furthermore, Silao et al. (2019) reported that induced respiration is critical for morphogenesis during the catabolism of morphogenic amino acids, which is an important feature for *C. albicans* to evade macrophages. *C. albicans* cells are highly adaptive to the inhibition of classical respiration; however, a recent study reported that a combination of AOX inhibitor salicylhydroxamic acid (SHAM) and sodium nitroprusside lead to fitness defects and the loss of viability in *C. albicans* (Duvenage et al., 2019). The thickness of the outer cell wall was reportedly thinner than that of untreated cells; however, no significant changes were observed in the relative levels of cell wall components including chitin, glucan, or mannan, suggesting that the inhibition of classical and alternative modes of respiration in *C. albicans* lead to organizational changes rather than the relative levels of cell wall components.

## AOX and Drug Resistance

The respiratory chain is an effective target for fungicides to control fungal contamination in food crops. Quinone outside inhibiting (QoI) fungicides represent the most important group of fungicides developed on the basis of mitochondrial inhibition (Bartlett et al., 2002). QoI fungicides inhibit fungal pathogens by blocking the transfer of electrons at the quinone outer binding site of the mitochondrial complex III. Development of resistance to QoI fungicides in fungal cells is a growing issue (Fernández-Ortuño et al., 2008). The acquisition of QoI resistance among numerous pathogens results from mutations in their cytochrome *b* or cytochrome *c* genes (Sierotzki et al., 2000). However, the most damaging emerging resistance to QoI fungicides is associated with AOX, which offers fungal cells the alternative respiration pathway that can bypass the target site for QoI fungicides. AOX leads to the flow of excess electrons when the cytochrome respiratory chain is inhibited or saturated, thus increasing the metabolic flexibility of fungal cells when exposed to biotic or abiotic stress potentially limiting the activity of the respiratory pathway (Juarez et al., 2006; Xu et al., 2012).

Along with ATP production, fungal mitochondrial function is associated with other important cellular functions including ergosterol biosynthesis and cell wall maintenance (Dagley et al., 2011). Specific inhibitors of fungal respiratory metabolism can reverse azole resistance (Vincent et al., 2016) and increase the sensitivity to fluconazole in *C. albicans* (Guo et al., 2014). Furthermore, AOX potentially contributes to fluconazole resistance in *C. albicans*, as combinatorial treatment with SHAM and fluconazole resulted in synergistic antifungal activity (Yan et al., 2009). Decreased ATP production may inhibit the activity of drug efflux pumps, thus decreasing drug resistance among fungal cells.

## AOX and Aflatoxin Production

Current methods of controlling aflatoxin contamination in food primarily depend on chemical and physical approaches usually focused on inhibiting the development of spores and mycelia, and/or inactivation of aflatoxins by their transformation to non-toxic compounds. Commonly used methods include the use of synthetic fungicides, irradiation, ozone fumigation, dehulling or cooking processes, regulation of environmental factors during harvest and storage, and the introduction of non-aflatoxin-producing *A. flavus* into the field to compete with the naturally occurring aflatoxin-producing strains (Ehrlich et al., 2014; Udomkun et al., 2017). These strategies are usually expensive, time-consuming, and inefficient, and some of them majorly alter the physical properties of food and cause serious loss of nutritive value; therefore, they are inappropriate for the elimination of aflatoxins from food. Synthetic fungicides are still the most widely used recourse to prevent fungal contamination of food crops. However, in addition to strict regulations regarding the use of synthetic compounds in food, the application of synthetic fungicides may result in notable drug resistance and serious environmental and health issues (Panáček et al., 2009). Awareness of these issues has led to an urgent need to develop novel antifungal and antiaflatoxigenic strategies.

Despite a dearth of knowledge of how AOX affects aflatoxin biosynthesis, AOX activity seems to affect sterigmatocystin, the penultimate intermediate in the biosynthesis of aflatoxin B_1_. Molnár et al. (2018a) investigated the association between AOX and sterigmatocystin synthesis in *A. nidulans* by both deleting and overexpressing the gene encoding AOX. Compared with the wild-type, the overexpressing mutant produced up to 70% more sterigmatocystin and the deletion mutant produced 50% less sterigmatocystin when grown in the dark. However, when the cultures were illuminated, sterigmatocystin productions were greatly reduced and exhibited no significant difference among the wild-type and the mutants. However, Leiter et al. (2016) reported that both the deletion and overexpression of AOX in *A. nidulans* negatively affected sterigmatocystin production. Nevertheless, these observations clearly indicate the importance of AOX in the regulation of sterigmatocystin and aflatoxin production.

Alternative oxidase potentially affects aflatoxin biosynthesis through the following different mechanism: (1) regulating fungal development; (2) redirecting cellular metabolism; (3) sustaining ATP and NADPH supply; and (4) maintaining cellular ROS levels. Most fungal secondary metabolites, including aflatoxin, are produced after the fungus completes its initial growth phase and is beginning a stage of development, represented by sporulation (Calvo et al., 2002; Amare and Keller, 2014). AOX is suggested to contribute to fungal physiology, morphology, and development (Osiewacz, 2011). AOX inhibitors have reportedly prevented *M. perniciosa*, *M. grisea*, and *Botrytis cinerea* spore germination *in vitro* (Inoue et al., 2012; Barsottini et al., 2019), suggesting that AOX activity is a common feature needed for spore germination-related pathways in these fungi. A previous study reported that on initiation of aflatoxin production, the external carbon source was greatly consumed, thus resulting in aflatoxin biosynthesis from the breakdown of reserve carbon sources such as lipids and fatty acids (Molnár et al., 2018a). AOX overexpression in *Podospora anserine* increased fatty acid production and decreased 2-oxoglutarate concentrations, suggesting a redirection in cellular metabolism (Bovier et al., 2014). It was hence suggested that AOX is accessory for increased aflatoxin production fueled by reserve lipids in the late stationary phase of growth. Meanwhile, it has been suggested that lipid molecules were crucial signals affecting the interaction between plant cells and mycotoxigenic fungi (Christensen and Kolomiets, 2011; Giorni et al., 2015). Studies on *A. flavus* have revealed the important role of oxylipins in the regulation of aflatoxin biosynthesis, conidia production, and sclerotia formation (Amaike and Keller, 2011; Scarpari et al., 2014). Thus, AOX, by modulating lipid metabolism, may also play an important role in regulating these fungal activities. Furthermore, aflatoxin synthesis requires considerable amounts of ATP and NADPH (Yabe and Nakajima, 2004). However, after the initial growth phase, the energy demand of fungal cells is reduced and carbon catabolism is inhibited through oxidative phosphorylation (Cárdenas-Monroy et al., 2017; Molnár et al., 2018a). To maintain cellular homeostasis, fungal cells may rely on alternative respiratory pathways for reoxidation of NADH for aflatoxin synthesis without concomitant ATP production. In addition, AOX activity may also affect aflatoxin production via a ROS-related mechanism. Previous studies have shown that aflatoxin biosynthesis implies a boost in oxygen uptake followed by an increase of ROS generation. This change occurred at the turning point between trophophase and idiophase when different secondary metabolites began to be prevalently produced (Zaccaria et al., 2015). AOX was also found to affect oxygen uptake in *Aspergillus* spp. (Table 1). It has been reported in many studies that ROS-induced oxidative stress stimulates aflatoxin production (Reverberi et al., 2008; Roze et al., 2015; Fountain et al., 2016; Umesha et al., 2017). The presence of multiple cytochrome p450 monooxygenases and monooxygenases in the aflatoxin biosynthesis pathway suggest the occurrence of both oxygen consumption and ROS production in this system.

## AOX Inhibitors and Their Application

Although alternative respiration produces markedly lesser ATP and appears dispensable for virulence in some fungal pathogens (Grahl et al., 2015), it maintains respiration and essential metabolic functions of the mitochondria in fungal cells when the classical electron transport chain is inhibited, thus enhancing fungal growth and viability. Therefore, a combination of classical and alternative respiration inhibitors is potentially the most effective strategy to control fungal contamination and limit the development of resistance (Duvenage et al., 2019). AOX inhibitors potentially exert synergistic antifungal effects with classical respiration inhibitors and other antifungal agents, which induce oxidative stress. However, owing to the lack of highly effective and fungal-specific AOX inhibitors, such a combination has not yet been tested *in vivo*.

Alternative oxidase of human parasite *Trypanosoma brucei* is currently the only alternative oxidase protein structure available (Shiba et al., 2013). AOX inhibitors appear promising for treating trypanosomiasis. The discovery of antibiotic ascofuranone and the optimization of existing inhibitors were indeed stronger AOX inhibitors and controlled trypanosome infections at very low doses (Ott et al., 2006; Ebiloma et al., 2019). The development of effective AOX inhibitors has received increasing interest. Unfortunately, owing to their low efficiency and selectivity, no advanced AOX inhibitors for practical, clinical and agricultural application have been reported. Our current understanding regarding the biological connections between AOX and the classical respiration of fungal cell has raised concerns that specific inhibitors of AOX may limit effects on the mitochondrial respiration, and thus cannot inhibit fungal propagation efficiently. However, Barsottini et al. (2019) reported a novel AOX inhibitor of optimized N-phenylbenzamide derivative that has strong AOX inhibitory effects, potentially prevents spore germination in the phytopathogen *M. perniciosa in vitro* and alleviates witches’ broom disease in infected plants. It suggests that further studies on the structure and physiological activity of AOX in fungal cells and the structure-activity relationship of current AOX inhibitors will surely promote the development of effective fungal AOX inhibitors to control fungal propagation. Besides, fungal mitochondrial function is associated with other important cellular functions including ergosterol biosynthesis and cell wall maintenance (Dagley et al., 2011), suggesting that respiration inhibitors potentially enhance the effects of current fungicides targeting those cellular phenomena. AOX is suggested to contribute to fluconazole resistance in *C. albicans*, as combinatorial treatment with SHAM and fluconazole displayed synergistic antifungal activity (Yan et al., 2009). The links between fungal cell walls and respiration are currently unclear. Nevertheless, combinatorial treatment with AOX and complex III inhibitors enhances the susceptibility of *C. parapsilosis* to caspofungin, a fungicide functioning by inhibiting β-D-glucan synthase (Chamilos et al., 2006). Furthermore, Duvenage et al. (2019) reported that the inhibition of classical and alternative respiration in *C. albicans* lead to changes in cell wall organization; however, the authors reported decreased susceptibility to caspofungin, highlighting the requirement for a deeper understanding of the physiological functions of fungal AOX and fungal mitochondria. Nevertheless, these studies indicated that AOX inhibitors, though they may be not efficient antifungal agents when working alone, still can contribute to fungal inhibition by working with current antifungal agents.

Another concern regarding the conservation of the respiratory machinery in pathogenic fungi and plants has also deterred the application of AOX inhibitors, since these inhibitors also act on plant AOX potentially and yield unexpected results. AOX is applicable in developmental plasticity and is associated with yield stability of food crops (Selinski et al., 2018; Barsottini et al., 2019). During soybean and cocklebur seed germination, AOX is associated with germination initiation, seedling growth, and chlorophyll synthesis. The inhibitory effect of SHAM on rooting has been observed among olives (Macedo et al., 2012; Porfirio et al., 2016). This highlights the requirement for AOX inhibitors that selectively act on fungal pathogens without disrupting normal plant activity, if AOX inhibitors are to be developed as successful antifungal agents to secure food production. Barsottini et al. (2019) presented the first study on the development of novel fungal AOX inhibitors and reported that an N-Phenylbenzamide derivative is more potent and selective than SHAM and inhibits *M. perniciosa* spore germination and prevents the appearance of the symptoms of witches’ broom disease in infected plants without obvious effects on the plant itself. This study suggests the possibility of developing AOX inhibitors acting specifically on fungal cells. Further studies on the structure and physiological differences of AOX in fungal and plant cells will surely promote the development of novel fungal-specific AOX inhibitors. Another strategy to ease this concern is to use natural compounds of vegetal origin, such as flavone, quercetin, resveratrol, and curcumin, which can potentially inhibit fungal respiration. These natural compounds modulate mitochondrial function through different methods including the inhibition of mitochondrial enzymes, suppression of oxidative phosphorylation, and alteration of the mitochondrial redox balance (Basile et al., 2009; Gibellini et al., 2010). In *Pichia stipitis*, and *M. grisea*, AOX induction by QoI fungicide was suppressed by flavonoid components (Wood and Hollomon, 2003). In *B. cinerea*, flavone reportedly inhibited respiration in whole cells treated with potassium cyanide. It is reasonable to expect that some plant-based natural compounds may inhibit fungal AOX without affecting plant cells. Alternatively, plant-based inhibitors of the classical respiration complexes might function synergistically with synthetic AOX inhibitors, thus effectively inhibiting fungal respiration with minimal or no impact on plant cells.

## Conclusion

In conclusion, fungal virulence composite AOX is potentially a suitable target owing to its association with pathogenesis, morphogenesis, environment adaption, fungicide resistance, cell wall regulation, lipid metabolism, and probably mycotoxin metabolism in studies on aflatoxin contamination and the propagation of *A. flavus*. AOX inhibitors, either alone or along with current antifungal agents, are potentially applicable for developing novel effective antifungal strategies. However, the application of AOX inhibitors in food production is currently limited by the low efficiency and selectivity, and concerns raised from the conservation of AOX in fungal and plant cells. To overcome these limitations, a deeper understanding of fungal alternative respiration and fungal AOX structure, and screening of effective fungal-specific AOX inhibitors are required.

## Author Contributions

FT, SL, and SW curated all references. FT drafted the manuscript. HC revised and finalized the manuscript. All authors read and approved the final version of the manuscript.

## Conflict of Interest

The authors declare that the research was conducted in the absence of any commercial or financial relationships that could be construed as a potential conflict of interest.
